# Occurrence of *Toxoplasma gondii* in raw goat, sheep, and camel milk in Upper Egypt

**DOI:** 10.14202/vetworld.2018.1262-1265

**Published:** 2018-09-15

**Authors:** Nagah M. Saad, Asmaa A. A. Hussein, Rania M. Ewida

**Affiliations:** 1 Department of Food Hygiene (Milk Hygiene), Faculty of Veterinary Medicine, Assiut University, Asyut, Egypt; 2 Department of Animal Hygiene and Zoonoses, Faculty of Veterinary Medicine, Assiut University, Asyut, Egypt; 3 Department of Food Hygiene (Milk Hygiene), Faculty of Veterinary Medicine, New Valley Branch, Assiut University, Asyut, Egypt

**Keywords:** camel milk, enzyme-linked immunosorbent assay, goat milk, quantitative polymerase chain reaction, sheep milk, *Toxoplasma gondii*

## Abstract

**Background and Aim:**

Toxoplasmosis is a worldwide zoonotic disease with harmful effects on animal and human health. Ingestion of contaminated raw milk has been suggested as a vehicle for transmission of *Toxoplasma gondii* to human. The present study was performed for the detection of *T. gondii* in raw milk samples of goat, sheep, and camel in Upper Egypt using two different techniques (enzyme-linked immunosorbent assay [ELISA] and quantitative polymerase chain reaction [qPCR]).

**Materials and Methods:**

This study was conducted to determine the *T. gondii* prevalence using ELISA and qPCR in raw goat, sheep, and camels milk (30 samples for each) collected from different locations in Upper Egypt.

**Results:**

*T. gondii* IgG antibodies were detected in 90.0, 60.0, and 3.33% of goat, sheep, and camel milk samples, respectively. From the positive samples of *T. gondii* IgG, the parasitic DNA was detected only in two examined milk samples, one of them was present in goat milk sample and another one was found in sheep milk sample. On the other hand, the parasite was not detected in camels’ milk samples.

**Conclusion:**

These results concluded that the raw milk was contaminated by *T. gondii* tachyzoites which could be a source of human infection. Restricted hygienic programs should be implemented in the animal farms to decrease the risk of milk contamination by this parasite.

## Introduction

Toxoplasmosis is one of the most important parasitic zoonoses of mammals and birds transmitted to human [1]. It affects approximately 25% of the human population [2]. This disease is caused by *Toxoplasma gondii*, an opportunistic protozoan belonging to *Apicomplexa* phylum [3]. Toxoplasmosis in human may occur vertically by passing the tachyzoites to the fetus through the placenta or horizontally by ingestion of foods contaminated by tachyzoites and bradyzoites as unpasteurized milk or cheese or uncooked meat or unwashed fruits/vegetables [4].

The clinical signs of toxoplasmosis in human ranged from abortion, stillbirth, and other congenital infections to eye disease in acute cases, while in chronic infection the fetal encephalitis is the most common sign [5]. Besides the clinical infection with *T. gondii*, 15-85% of the human population are asymptomatically infected with this parasite [6], the wide range of Toxoplasmosis infection based on the geographic location.

A few studies reported the presence of tachyzoites in milk of different species as goat, sheep, cow, buffalo, and camel [4,7-9]. Toxoplasmosis in goat and sheep has great significance because it leads to many economic and production losses which consequently transmitted to human [10].

There are many different diagnostic methods for *T. gondii* diagnosis, such as serological detection (enzyme-linked immunosorbent assay [ELISA] and latex agglutination test), cell line culture, bioassays, and molecular techniques [11]. Serological tests used more as a preliminary test to detect the parasite, while polymerase chain reaction (PCR) is the best choice for diagnosis of *T. gondii* infection [12] because it has shown higher accuracy, sensitivity, and specificity than the other diagnosis methods [13,14].

The occurrence of *T. gondii* in raw milk samples is essentially unknown, and the current study was performed for the detection of *T. gondii* in raw milk samples of goat, sheep, and camel in Upper Egypt using two different techniques (ELISA and quantitative PCR [qPCR]).

## Materials and Methods

### Ethical approval

Ethical approval is not required to pursue this type of study.

### Samples and study area

A total of 90 raw milk samples were collected from goat, sheep, and camels (30 samples for each) in different cities of Upper Egypt. Each milk sample (50 ml) was collected from the apparently healthy single animal in a sterile screw cap bottle aseptically. The sample was divided into two parts and they were stored at −20°C until ELISA analysis and DNA extraction. The lab work was done in the Molecular Biology Research Unit in Assiut University (Certified ISO/IEC: 17025-2005).

### ELISA assay

The first parts of the milk samples were prepared and examined as described in the manual kit of Toxoplasma IgG Enzyme Immunoassay Test Kit (Precheck Bio, USA). The positive samples were subjected to confirmation using quantitative polymerase chain reaction (qPCR).

### qPCR

DNA extraction was carried out using Patho Gene-spin^™^ DNA/RNA Extraction kit (iNtRON Biotechnology, Korea) according to manufacturer’s instruction. The extracted DNA was stored at −20°C.

DNA amplification was carried out using Primerdesign^™^ Ltd., and *T. gondii* repeat region genesig^®^ Standard kit and oasig^™^ lyophilized 2× qPCR Mastermix (Uk) qPCR were performed for DNA amplification in final volume 20 μl (10 μl of oasig^™^ 2× qPCR master mix, 1 μl primer/probe mix, 1 μl of each primer, 5 μl DNA template [25 ng], and up to 4 μl Nuclease-free water were mixed in a PCR tube).

The amplification was performed in Mx3000P platforms (Stratagene, Germany) at 95°C for 2 min, followed by 50 cycles under the following conditions; denaturation at 95°C for 10 s, data collection at 60°C for 1 min.

### Statistical analysis

The incidence of *T. gondii* IgG in the examined raw milk samples was calculated by dividing the number of seropositive samples by the total number of the examined samples, while, the frequency distribution of *T. gondii* in the examined raw milk samples using qPCR was calculated by dividing the positive sample of DNA by the total number of the seropositive samples. Data were entered into Microsoft Excel Spreadsheet.

## Results

Serological prevalence of *T. gondi*i IgG: The seropositive samples were distributed as follows 27 samples (90%) of goat milk samples, followed by 18 samples (60%) of sheep milk samples, while IgG of *T. gondii* was detected in one sample only (3.33%) from the examined camel milk samples (Table-1).

**Table-1:** Incidence of *T. gondii* IgG in the examined raw milk samples.

Type of milk	No. of examined samples	Positive of ELISA test

		n (%)
Goat milk	30	27 (90)
Sheep milk	30	18 (60)
Camel milk	30	1 (3.33)
Total	90	46 (51.11)

*T. gondii*=*Toxoplasma gondii*, ELISA=Enzyme-linked immunosorbent assay

Molecular detection using qPCR: The seropositive samples were subjected to further examination using qPCR. The DNA of *T. gondii* was detected in only two raw goat and sheep milk samples (one for each) from total 46 samples that were positive in ELISA test (Table-2).

**Table-2:** Frequency distribution of *T. gondii* in the examined raw milk samples using qPCR.

Type of milk	No. of examined samples	Positive of qPCR test

		n (%)
Goat milk	27	1 (3.70)
Sheep milk	18	1 (5.55)
Camel milk	1	- (0)

*T. gondii*=*Toxoplasma gondii*, qPCR=Quantitative polymerase chain reaction

## Discussion

Consumption of unpasteurized milk and its products represents many public health risks including transmission the tachyzoites of *T. gondii* to the human. The tachyzoites may be destroyed by gastric juice [7], while other evidence indicates that the gastric juice could not destroy the tachyzoites and it can cause infection to human [15]. Unpasteurized or inadequately processed milk or fresh cheese is the most important food in rural areas, which can be a high significance means of contamination by *T. gondii* [16].

In the present study, a preliminary survey of *T. gondii* on milk samples collected from goat, sheep, and camels using ELISA techniques was conducted. The highest incidence of *T. gondii* IgG in the examined samples was goat milk samples (90%). This result of the present study was higher than the result obtained by Abdel-Rahman *et al*. [17], Sadek *et al*. [18], and Mosa [19]. Furthermore, the incidence of antibodies of *T. gondii* in the examined sheep milk samples was higher than the results carried out by Sadek *et al*. [18] and Mosa [19] who reported that the presence of antibodies in the sheep milk samples in Egypt was 39.66 and 14.29%, respectively. Moreover, the lowest seroprevalence of *T. gondii* found in the analyzed camels’ milk samples, and this finding is nearly similar to Dehkordi *et al*. [8] results, they stated that the prevalence of *T. gondii* antibodies in camel milk samples was 3.12% in Iran.

The higher incidence of *T. gondii* in goat and sheep milk rather than the camel milk is due to the difference between the types of pastures of small ruminates and the camels. The goat and sheep feeding on green pastures which contaminated by cats’ feces which are the source of oocytes of *T*. *gondii* [20,21]. While the camels mainly feed on the dry feed which presents in the desert and this feed fairway from the cats’ feces which is the main source of *Toxoplasm*a oocytes.

**Figure-1:**
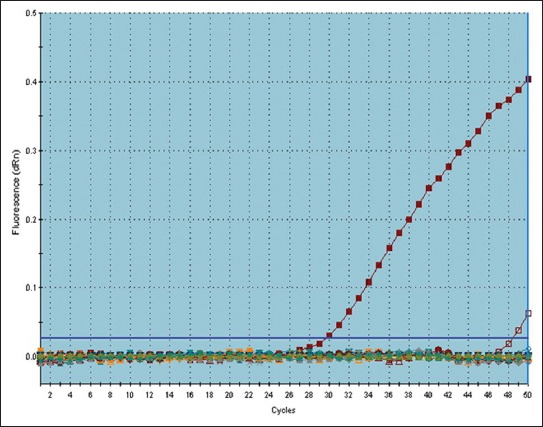
Amplification plots of *Toxoplasma gondii* in goat milk samples. The first plot indicates the positive control with CT 29.73; the second amplified curve indicates one of the positive samples with CT 48.48 and the other samples not amplified and they are running under the threshold.

Molecular techniques are more accurate, sensitive, and specific assays than the serological techniques because it can detect the DNA of the parasite, not the antibodies produced against *T. gondii*. The finding in this study revealed that only two samples from goat and sheep milk (one of each) contained DNA of *T. gondii*., as shown in Table-2 and Figure-1. However, the DNA of the parasite failed to be detected in camel milk samples. These results were lower than the obtained by Sadek *et al*. [18], Mosa [19], and Ossani *et al*. [22]. The low numbers of incidence of *T. gondii* detected by qPCR in comparison with antibodies of Toxoplasma are due to the fact that IgG antibodies are produced in the late stage of infection, and the parasite is being in localized in the organs and tissues and not circulated in the blood, thus finally it will not reach to the milk.

Many different molecular techniques are used for diagnosis of *T. gondii* in milk samples as conventional PCR [18,19], nested PCR [23], PCR-RFLP technique [24], and qPCR [25,26]. qPCR assay is the most rapid, accurate, and effective molecular technique that can be used for *Toxoplasma* detection [27,28].

## Conclusion

This study detected the presence of *T. gondii* in the raw goat, sheep, and camels milk samples obtained from different locations in Upper Egypt which could lead to infection of human by ingesting this contaminated milk and its products. In addition, preventing the entrance of street cats in the farms to avoid contamination of animals’ feeds by the cats’ feces. Moreover, periodical examination of animals’ milk using serological and molecular assays help in monitoring the diseases and thus be helpful to control the spreading the infection among animals and human.

## Authors’ Contributions

NMS, AAAH, and RME designed the plan of work experiment. RME carried out the laboratory work. All authors analyzed the results, drafted, read, and approved the final manuscript.
